# Health care strategy for ensuring work ability in an aging Korea

**DOI:** 10.1186/s40557-016-0127-y

**Published:** 2016-09-07

**Authors:** Jungsun Park, Jong-tae Park, Soo Geun Kim, Cheol-In Yoo, Junseok Son, Jun Yim, Dae-seong Kim, Kyung Young Rhee, Yangho Kim

**Affiliations:** 1 Department of Occupational Health, Catholic University of Daegu, Gyongsan, South Korea; 2 Department of Occupational Medicine, Korea University Ansan Hospital, Ansan, South Korea; 3 Department of Occupational Medicine, Kangbuk Samsung Hospital, Sungkyunkwan University, School of Medicine, Seoul, South Korea; 4 Department of Occupational and Environmental Medicine, Ulsan University Hospital, University of Ulsan College of Medicine, Ulsan, South Korea; 5 Department of Occupational & Environment medicine, Samsung Changwon Hospital, Sungkyunkwan University School of medicine, Changwon, South Korea; 6 Department of Preventive Medicine, Gachon University College of Medicine, Incheon, South Korea; 7 Occupational Safety and Health Research Institute, Korea Occupational Safety and Health Agency, Ulsan, South Korea

**Keywords:** Work ability, Aging, Health promotion

## Abstract

The rapid aging trend in South Korea will cause a growing shortage of labor and decreasing quality of the labor force. The purpose of this commentary is to recommend a health care strategy to maintain and promote the work ability of employees in an aging Korea. Strategies to promote the work ability require the collaboration of governmental agencies at the central and local levels. First, the common goal should be the reinforcement of follow-up measure in general medical examinations and the promotion of healthy lifestyles for workers. Second, collaborating activities should be performed among the Worker’s Health Center, the Health Promotion Center, and community health centers. In conclusion, health care strategies for ensuring the work ability in an aging Korea require the collaboration of governmental agencies at the central and local levels.

## Background

South Korea’s population is aging more rapidly than any other country [1, 2]. Since it became an aging society in 2000, the predictions are that South Korea will be an aged society by 2018 and a super-aged society by 2026 [1]. Labor shortages and a lack of skilled people are expected due to a reduction in the size of the productive population (age 15–64) in 2017 and the mass retirement of “baby boomers” [1]. Further, the most economically active age group (30–49 years-old) in the productive population will decline continuously, from 49 % in 2005, to 42 % in 2020, and to 37 % by 2050. Similarly, the percentage of elderly people (at least 50 years-old) was 25 % in 2010, and is expected to exceed 33 % in 2020 [1, 3]. Thus, the aging of the productive population will quickly reach the point when most current baby boomers have left the labor market (after 2030).

This aging of the workforce will likely increase the accident rate because the elderly experience degradations of many physiological functions, such as sensory systems, sense of equilibrium, and motor control [4, 5]. In addition, the increased prevalence of chronic and degenerative diseases due to aging will lead to a decreased quality of the labor force [6].

Thus, it is clear that the rapid aging trend in South Korea will have a significant impact on the quantity and quality of labor. Therefore, the purpose of this commentary is to recommend a health care strategy to maintain and promote the work ability of employees in an aging Korea.

### The current health care system for employees in South Korea

The government’s comprehensive plan, ‘The Second Plan for Aging Society and Population (2011-2015)’, lacked life-cycle-specific health care measures (i.e. measures for young and middle-aged workers) in the ‘proactive health care system’ section [7]. It also placed the public health center for the community population at the center of most activities, including improvements in health screening and follow-up systems, establishment of a community-based integrated health care system, institutionalization of health care services, and introduction of chronic degenerative diseases management programs. Even though the Framework Act on Health and Medical Services Article 36 (Occupational Health Care) states that the government should take measures to protect and promote employees’ health [8], almost none of these activities have been carried out for the employees, the most important target for management of chronic degenerative diseases.

Based on the current Occupational Safety and Health Act, workers' medical examinations are to be carried out regularly in all workplaces, regardless of business size. However, workplaces with fewer than 50 workers do not have professional personnel to perform follow-up measures after medical examinations, and this is a significant problem in the current workers’ health care policy. In addition, workplaces with fewer than 2000 workers (or fewer than 3000 or 5000 workers in some sectors) are not required to appoint a doctor or nurse according to the Occupational Safety and Health Act [9]. Thus, a number of workplaces rely on external expertise for workers’ health care services, such as follow-up measures of periodic medical examinations. Follow-up measures for general medical examinations tend to be neglected compared to occupational medical examinations, although employers are required to perform these follow-ups. In addition, workplaces and external professional organizations for occupational health programs lack specialized personnel, facilities, and equipment for health promotion and health promotion programs. Thus, establishment of a linkage with health promotion resources in the community is urgently needed.

### Benchmark: strategies to promote work ability in aging Japan

Japan has guidelines on cooperation between community health and occupational health activities for workers’ health promotion at the level of the local community, and has performed cooperative activities since 2005 [10]. The implications of these policies and guidelines is as follows. First, the law should have a provision for harmonization and collaboration of existing health services that were previously carried out independently in different department of one ministry. Second, it is necessary to have common recognition of the collaborative activities. Each agency holds the value of the existing independent business, so it is necessary to share the recognition of the need and significance of collaborative activities for the community’s health problems through the exchange of information between institutions regarding health information and services. Third, it is important to establish guidelines for collaborative activities so there is a clear and mutual understanding and a systematic project plan based on the consensus. Lastly, it is crucial to establish a local council to promote these collaborative activities.

### Recommendation for policy

We described in detail a process leading to a recommendation for promoting work ability in an aging society in a Table, which shows a logic for solution containing current status and problem identification, counter-measures for the problem, obstacles, and solution (Table 1).

**Table 1:** Process leading to a recommendation for promoting work ability in an aging society

Current status and problem	Low birth rate and aging population
	⤓
	Aging of the workforce
	⤓
	Shortage of labor/Reduced work ability
	<Cause 1> The high prevalence rate of chronic degenerative diseases in the elderly, such as hypertension and diabetes mellitus, causes an increase in the number of people with cerebrovascular and cardiovascular disease.
	(※ From now on, chronic degenerative diseases (*e.g.* cerebrovascular and cardiovascular disease, degenerative arthritis, and depression) will be a major occupational health issue that have a greater impact on quality of life and worker productivity than occupational diseases.)
	< Cause 2> There is an increase in accidents due to physiological deterioration of workers such as visual/auditory function, muscle strength, and body control.
Counter-measure	1. Prevention of cerebrovascular and cardiovascular disease in workers.
	<Strategy> To conduct follow-up management based on risk level through assessment of cerebrovascular and cardiovascular diseases from general health examinations.
	- Moderate- and high-risk groups: strict management of basic diseases such as hypertension and diabetes.
	- All groups: reduction of risk factors based on lifestyle, such as smoking, unhealthy dietary habits, and lack of physical activity, through workers’ health promotion programs.
	2. Plan to improve the work ability of the elderly
	< Strategy> Support the establishment of a work environment that is appropriate for elderly workers who have reduced physical capacity.
Obstacles	1. Employers do not consider cerebrovascular and cardiovascular disease prevention activities as a mandatory occupational health measure.
	2. General medical examination results are usually not accessible to employers, unlike the occupational medical examination results.
	3. Professional personnel running health promotion programs in the workplace are often not present or are unavailable.
Solutions	1. Massive publicity campaign on the significance of follow-up measures of general medical examinations and the prevention of cerebrovascular and cardiovascular diseases as employer obligations.
	2. National health examination data sharing between the Ministry of Health and Welfare (National Health Insurance Corporation) and the Ministry of Employment and Labor (Korea Occupational Safety and Health Agency).
	3. Establish and operate a local council and collaborate with local governments to make full use of community resources such as a public health center, health promotion center, and workers’ health center.
	(※ Ensuring stable funding for active workplace health promotion programs/Revising the relevant laws and regulations, such as National Health Promotion Act and Framework Act on Health Examination, to make it easier to use community resources.)

The policy for promotion of work ability in a low-birth rate and aging society must be included in the 4^th^ Five-Year Plan for Industrial Accident Prevention (2015–2019) [11]. The principle to implement this policy is as follows: First, prevention programs should be tailored to the age of the workers (adolescence, middle age, seniors, etc.). Second, there should be prevention programs through collaboration among the relevant ministries (Ministry of Employment and Labor/Ministry of Health and Welfare/Ministry of Culture, Sports and Tourism, etc.). Third, prevention programs should be linked to community resources. The management of chronic degenerative diseases, using follow-up measure in general medical examinations, should be placed at the center of these principles.

The principles and strategies to promote work ability through the collaboration of relevant agencies at the central and local level is as follows. A common goal–the reinforcement of follow-up measures of general medical examinations and the promotion of healthy lifestyles for workers–must be set up by signing a memorandum of understanding between the Ministry of Employment and Labor and the Department of Health and Welfare. In addition, the Ministry of Employment and Labor should inform all employers of the significance of follow-up measures of general medical examinations. Once this basic governmental policy is announced, workers’ health promotion activities by employers will gain momentum, conditions for collaboration will be established, and an environment for collaboration will be created that naturally leads to collaboration.

**Fig. 1:**
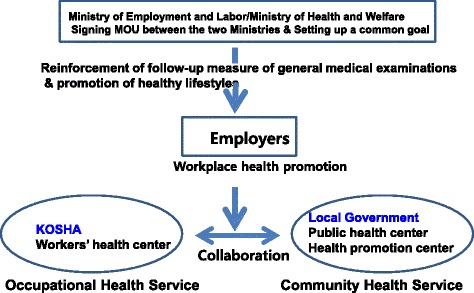
Reinforcement of workplace health promotion activities through the collaboration of central and local agencies in Korea

In order to promote cooperation between community health and occupational health activities for health promotion in workers in Korea, several points should be addressed (Fig. 1). First, workers’ health information should be shared between the National Health Insurance Corporation and the Korea Occupational Safety and Health Agency. Second, a standard code should be presented to share data by linking the websites that each agency operates to provide integrated information. Third, when setting up the cooperation, the target institutions should be the Korea Occupational Safety and Health Agency, the National Health Insurance Corporation, and the local government at the administrative agency level. The active organizations should be the Worker’s Health Center, the Health Promotion Center, and the community health centers, respectively, at the local activity level. Finally, there should be a steering team to promote employees’ work ability in the Ministry of Employment and Labor. Under the leadership of the Ministry of Employment and Labor, it will play the role of a “control tower” to promote collaboration, and form a group consisting of about 10 individuals below the vice Minister who operate together. This should include all institutions participating in the collaboration, and should hold periodical conferences to monitor the process and results.

## Conclusions

Health care strategies to promote the workability in an aging Korea require the collaboration of governmental agencies at the central and local levels.
